# A photobioreactor for production of algae biomass from gaseous emissions of an animal house

**DOI:** 10.1007/s00253-023-12815-7

**Published:** 2023-10-10

**Authors:** Till Glockow, Marta Velaz Martín, Laura Meisch, Denis Kapieske, Kai Meissner, Maximiano Correa Cassal, Anne-Kristin Kaster, Kersten S. Rabe, Christof M. Niemeyer

**Affiliations:** 1 Acheron GmbH, Auf der Muggenburg 30, 28217 Bremen, Germany; 2 Institute for Biological Interfaces 1 (IBG-1), Biomolecular Micro- and Nanostructures, Karlsruhe Institute of Technology (KIT), Hermann-von-Helmholtz-Platz 1, 76344 Eggenstein-Leopoldshafen, Germany; 3 Institute for Biological Interfaces 5 (IBG-5), Biotechnology and Microbial Genetics, Karlsruhe Institute of Technology (KIT), Hermann-von-Helmholtz-Platz 1, 76344 Eggenstein-Leopoldshafen, Germany

**Keywords:** Animal agriculture, Ammonium sequestration, Photobioreactor, *Spirulina*

## Abstract

**Abstract:**

Sustainable approaches to circular economy in animal agriculture are still poorly developed. Here, we report an approach to reduce gaseous emissions of CO_2_ and NH_3_ from animal housing while simultaneously using them to produce value-added biomass. To this end, a cone-shaped, helical photobioreactor was developed that can be integrated into animal housing by being freely suspended, thereby combining a small footprint with a physically robust design. The photobioreactor was coupled with the exhaust air of a chicken house to allow continuous cultivation of a mixed culture of *Arthrospira spec.* (*Spirulina*). Continuous quantification of CO_2_ and NH_3_ concentration showed that the coupled algae reactor effectively purifies the exhaust air from the chicken house while producing algal biomass. Typical production rates of greater than 0.3 g/l*day dry mass were obtained, and continuous operation was possible for several weeks. Morphological, biochemical, and genomic characterization of *Spirulina* cultures yielded insights into the dynamics and metabolic processes of the microbial community. We anticipate that further optimization of this approach will provide new opportunities for the generation of value-added products from gaseous CO_2_ and NH_3_ waste emissions, linking resource-efficient production of microalgae with simultaneous sequestration of animal emissions.

**Key points:**

*• Coupling a bioreactor with exhaust gases of chicken coop for production of biomass.*

*• Spirulina mixed culture removes CO*_*2*_ *and NH*_*3*_ *from chicken house emissions.*

*• High growth rates and biodiversity adaptation for nitrogen metabolism.*

**Graphical abstract:**

Towards a sustainable circular economy in livestock farming. The functional coupling of a helical tube photobioreactor with exhaust air from a chicken house enabled the efficient cultivation of Spirulina microalgae while simultaneously sequestering the animals’ CO_2_ and NH_3_ emissions.

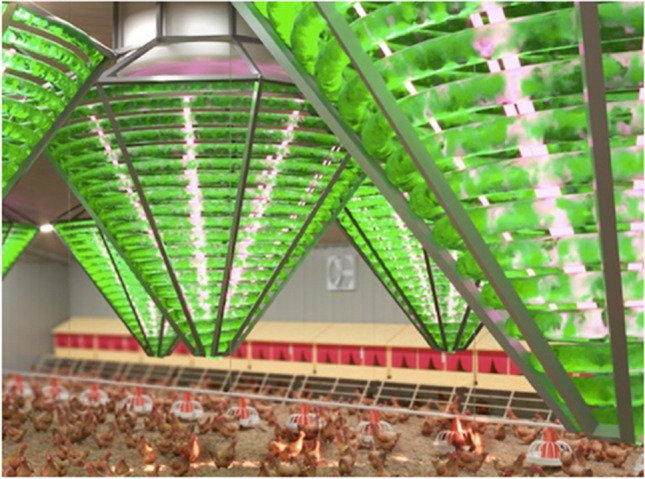

**Supplementary Information:**

The online version contains supplementary material available at 10.1007/s00253-023-12815-7.

## Introduction

As a result of the growing world population, the global demand for food is increasing, while at the same time, the available arable land is already exhausted and can only be expanded through progressive deforestation. Therefore, the development and provision of performance-increasing and resource-saving, sustainable agricultural production is essential for mankind (Lampridi et al. 2019). Closed-loop systems that allow highly efficient use of available land and raw materials within a production process would be ideal. Controlled environment agriculture (CEA) in urban areas is one of such approaches that addresses concerns about conventional agriculture and the increasing demand for food (Dsouza et al. 2021). Such urban CEA systems specifically include farming techniques like climate-controlled greenhouses and plant factories with artificial lighting that can provide high yields regardless of external environmental conditions. For example, a recent study suggested that vertical farming under optimized conditions could produce up to 600 times of the current global average annual wheat yield (Asseng et al. 2020).

In contrast to the advanced prospects in plant agriculture, sustainable approaches to circular farming in animal agriculture are less well developed. This branch of agriculture, which deals with animals raised for meat, fiber, milk, or other products, imposes a heavy burden on the environment worldwide (Andretta et al. 2021; Gržinić et al. 2023). In Germany alone, for example, pigs and poultry are kept on more than 200,000 farms, resulting not only in barely tolerable environmental pollution from feces and manure, but also in enormous emissions of CO_2_, NH_3_, and other volatile compounds. While solid and liquid waste emissions from animal husbandry can be converted into more valuable substances with the help of biogas plants (Abanades et al. 2022; Esteves et al. 2019), approaches to reduce gaseous emissions from animal houses and at the same time use them for the production of valuable substances are scarce.

The upgrading of CO_2_ to valuable compounds can be achieved by, e.g., photosynthetic microorganisms (PMO), such as eukaryotic (micro)algae or *Cyanobacteria*, which bind CO_2_ by photosynthesis and which are also able to filter nitrogen and sulfur compounds or other gaseous or aerosol-bound substances from the air. For their growth, these PMO require light, CO_2_, water, and inorganic nutrients such as nitrogen and phosphorus sources, as well as trace elements. Under optimal conditions, typically with temperatures in the range between 20 and 40 °C, a sufficient carbon source, and light energy, PMO can double their biomass within 12 h and convert large amounts of gaseous emissions into biogenic substances through photosynthesis. It is therefore not surprising that PMO cultivation is an established technology to produce valuable biomass in a resource-saving way by now (Fernandes et al. 2015). In PMO cultivation processes, carbon usually accounts for more than half of the dry weight of the biomass produced. However, the PMO cells are also rich in a wide variety of biogenic and bioactive substances, such as amino acids, proteins, carbohydrates, oligo- and polysaccharides, unsaturated fatty acids, and numerous secondary metabolites, such as vitamins, dyes, or antibiotics (Pierobon et al. 2018). Moreover, nowadays, the choice of PMO is not limited to naturally occurring strains, but with the help of genetic engineering, recombinant species are also accessible, producing certain biogenic constituents in high yields. This makes high-value products for energy, food, or health applications, such as enzymes or even therapeutic antibodies accessible (Ahmad et al. 2020).

PMO and other microorganisms are also used for bioremediation to remove pollutants from liquid, solid, and gaseous waste streams (Bala et al. 2022; Roy et al. 2022; Touliabah et al. 2022). For example, microorganisms on a solid support material can be used as powerful biofilters for air purification to remove air pollutants in exhaust gases (Hussain et al. 2021), and PMO are established for bioremediation of liquid media, such as to improve nitrogen waste management in recirculating aquaculture systems (Ramli et al. 2020). While the use of PMO for CO_2_ sequestration (Cheah et al. 2015; Kumar et al. 2011; Onyeaka et al. 2021) is an established approach as it allows for simultaneous air cleaning and biomass production, its application for reducing gas emissions from animal housing is not well studied.

Suitable photobioreactors, which could be used under the conditions given in conventional animal houses to utilize animal emissions (e.g., chemical components contained in the air) and waste heat from the animals for the production of valuable substances with the help of PMO, would have to meet certain requirements. The photobioreactor should be directly and easily integrated into existing animal barns. Since conventional barns require the entire floor space for the animals (pigs, cattle, sheep, poultry, etc.), a suitable device should have the smallest possible footprint and should have high physical robustness to avoid mechanical damage to the reactor and its operating elements (light fixtures, pumps, valves, etc.). In addition, a suitable photobioreactor should be scalable in terms of size and culture volume, and should be easily integrated into serial or parallel arrangements. Last but not least, the reactor should have the best possible performance in terms of PMO culture efficiency.

**Fig. 1:**
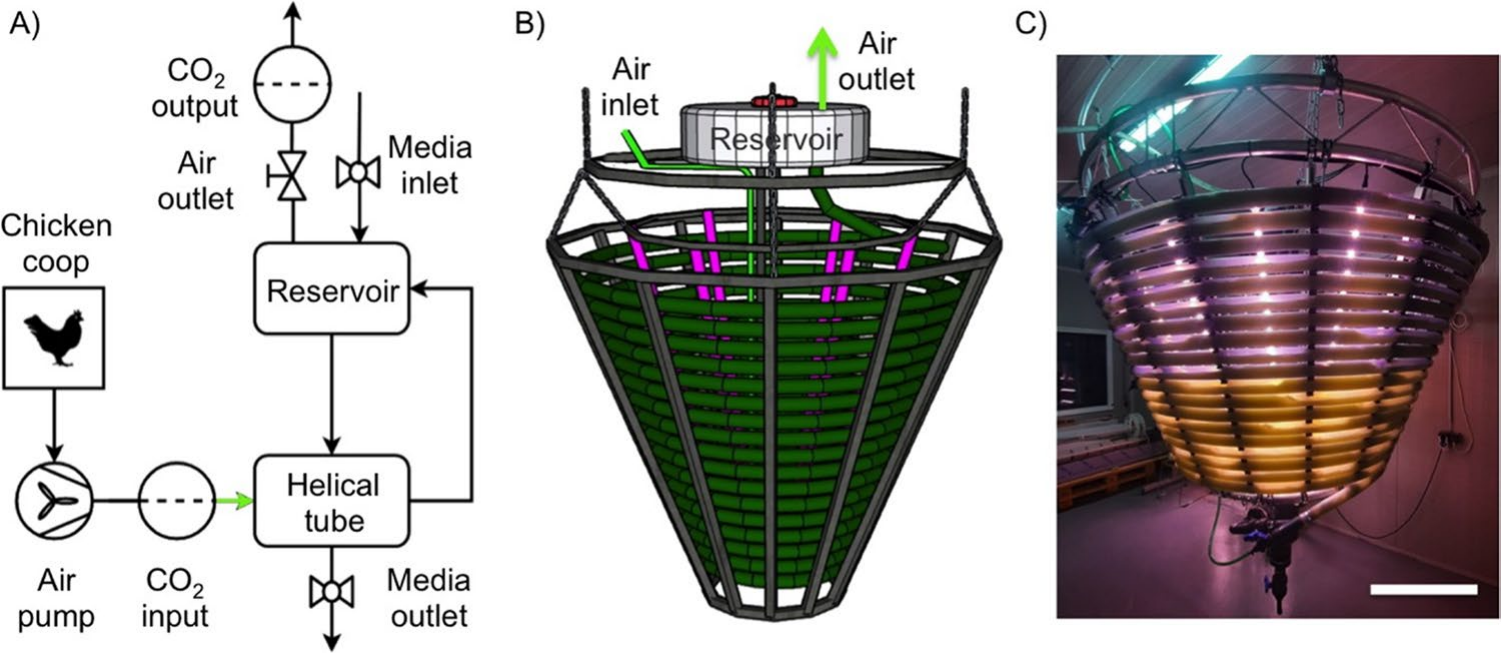
Details of the photobioreactor setup. **A** Schematic flow diagram and illustration of the coupling of the chicken coop with the photobioreactor. The exhaust air from the coop is taken by an air pump via a CO_2_ “input” sensor and blown into the bioreactor via the air inlet at the bottom, rises into the reservoir via the helical tube, and escapes via the gas outlet and the CO_2_ “output” sensor. The buoyancy of the rising air transports the medium through the helix into the reservoir, and then flows back from there through a central return tube into the lower part of the helix. **B** Technical drawing and **C** photographic image of the helical tube photobioreactor. Note that the reservoir in the prototype shown is located inside the helical tube and is therefore not visible. The LED light bars located inside the helix can be seen in pink. Scale bar is 500 mm

Of the almost infinite number of closed photobioreactors described in the literature (Kunjapur and Eldridge 2010), we chose a cone-shaped, helical tubular reactor (Watanabe and Hall 1996), as this allows combining a small footprint with a robust design when the reaction tube is made of transparent flexible tubing material and such a device is implemented in a suspended structure. We report here for the first time the performance of a prototype of such a suspended helical cone photobioreactor for reducing gas emissions from a chicken house to produce *Spirulina* (*Arthrospira spec.*), a cyanobacterium (Fig. 1). Quantification of CO_2_ and NH_3_ concentration showed that the coupled algae reactor effectively purifies the exhaust air from the chicken house while producing algal biomass. Continuous operation was possible for several weeks. Metagenomics analysis of the *Spirulina* culture showed that exhaust air from the chicken house altered the composition of the algal culture and provided clues to NH_3_ metabolism.

## Materials and methods

### Microalgae cultivation

Of the photosynthetic microorganisms (PMO) considered to be of commercial importance, species of the genus *Spirulina*, now called *Arthrospira spec.*, are among the most important (Gonçalves et al. 2016; Soni et al. 2019). In this study, a mixed culture of *Spirulina* microalgae (*Arthrospira platensis*), kindly provided from an open pond culture of a microalgae farm (ROVAL GmbH, Rockstedt, Germany), was used as a starter culture for continuous cultivation in closed photobioreactors. The culture medium was prepared from tap water supplemented with 1.45 g/l Agrolution 324 fertilizer (ICL Fertilizers, Ludwigshafen, Germany), 0.19 g/l KALISOP (K + S Aktiengesellschaft, Kassel, Germany), 0.033 g/l Micromax WS Fe Chelate (ICL Fertilizers), 1 g/l Canna Trace Mix Mono (CANNA GmbH, Kleve, Germany), 55 g/l sodium chloride (Südwestdeutsche Salzwerke AG, Heilbronn, Germany), and 9 g/l sodium bicarbonate (Höfer Chemie GmbH, Kleinblittersdorf, Germany) to obtain final concentrations of 203 mg/l N, 51 mg/l P, 344 mg/l K, 35 mg/l S, 4.3 mg/l Fe, and 17 mg/l Mg. To grow inoculum for the helical tube reactor, the starting culture obtained from ROVAL was first propagated in conventional 10 l algabags (Algatec, Sottrum, Germany) under aeration with regular air for about 10 days until the optical density at 565 nm was about 2.0. Thirty liters of this algae suspension together with 115 l of nutrient medium was filled into the helical tube reactor via the media inlet (Fig. 1) and aeration inside the reactor was started with an air supply of 15 l/min. Cultivation was performed in air-lift mode under continuous illumination with 15 LED lamps, 28 W each (L28 AP673L, VALOYA, Helsinki, Finland), installed inside the helical cone (see Fig. 1). The progress of algal growth was determined by sampling (see the “Analytics” section), and when the biomass of about 0.9–1 g dry weight per liter was reached (typically in 3–4 days), parts of the biomass were removed by rotational filtration. For this purpose, 90 l of algal suspension was taken through the reactor’s media outlet and filtered using a drum filter (Spirutech, Entrepierres, France). The harvested cleared media was post-fertilized with 720 g/kg (harvested dry weight) Agrolution 324, 65 g/kg KALISOP, 10 g/kg Micromax WS Fe chelate, 100 g/kg Canna Trace Mix Mono, and 2000 g/kg sodium bicarbonate and then returned to the helical tube reactor through the media inlet for the next growth cycle. When the growth rates observed in the cycle dropped (typically after 4–6 weeks), the entire long-term cultivation process was restarted with fresh inoculum as described above.

### Analytics

To determine growth, aliquots of the algal suspension were taken at regular intervals through the reactor outlet and the biomass contained therein was determined photometrically by absorbance measurement at 565 nm (HI801 Iris Photometer, Hanna Instruments, Vöhringen, Germany). To convert the absorbance values to dry mass, a calibration curve was prepared by measuring corresponding samples after vacuum filtration and drying in triplicates (Figure S1).

The pH of the aqueous solutions was determined using a pH meter (PH22 digital, PCE Deutschland GmbH, Meschede, Germany). For morphological characterization of algae, a light microscope (Ortholux II, Leitz, Wetzlar, Germany) was used. To determine the CO_2_ concentration in the exhaust air of the chicken house before (input CO_2_) and after (output CO_2_) passage through the algae reactor, T6545 meters (COMET SYSTEM, Bezručova, Czech Republic) were used. Data point intervals were every 15 min, and calibration to 400 ppm was performed for both instruments simultaneously on outside air as specified by the manufacturer. For the quantification of NH_3_, Hydrion Ammonia Test Paper strips (Micro Essential Laboratory Inc., New York City, USA) were moistened with distilled water according to the manufacturer’s instructions and placed in the gas stream for 15 s and read immediately from the color scale on the test kit (see Figure S2). To increase statistical confidence, these measurements were performed repeatedly over a longer period of time (> 30 days) and the individually obtained values (*n* > 10) were averaged.

### Sequencing analysis

For the extraction of genomic DNA (gDNA), 1 ml of algae suspension was collected in a 2 ml sterile Eppendorf tube. The suspension was centrifuged for 20 min at 16,000 × g to remove the excess of medium/water. By using the commercial DNeasy PowerSoil Pro Kit (QIAGEN, Hilden, Germany), the gDNA of the remaining pellet was extracted by following the manufacturer’s instructions. The concentration of extracted gDNA was quantified photometrically by NanoDrop OneC Microvolume and fluorometrically by Qubit 3 (both from Thermo Scientific Inc.). Metagenomics libraries from the purified gDNA were prepared by using the NEBNext® Ultra™ II FS DNA Library Pro Kit for Illumina® (New England Biolabs, Germany), after which the quality of the libraries was verified using the Agilent High Sensitivity DNA Kit on the Agilent 2100 Bioanalyzer instrument (Agilent Technologies, Germany). DNA libraries were sequenced on an Illumina NextSeq550® (New England Biolabs, Germany) device by using 300 cycles and a paired-end approach.

Raw sequences were processed to remove adapter sequences and low-quality reads using successively trimmomatic v.0.36 (Bolger et al. 2014), bbduk v.35.69 (Bushnell 2022), and cutadapt v.1.1.4 (Martin 2011). High-quality overlapping paired-end reads were merged using FLASH, v.1.2.11 (Magoc and Salzberg 2011). Assembly of the merged reads was performed with MEGAHIT v.1.2.9 (Li et al. 2015). Subsequently, coverage profiles were created by using samtools v.1.9 (Li et al. 2009) and BamM v.1.7.3 (Li and Durbin 2009). These profiles were used to perform taxonomic classification based on rRNA marker genes, which were identified by using the get_markers function of MDMcleaner (Vollmers et al. 2022) and classified with SINA v.1.7.2 (Pruesse et al. 2012). Prodigal v.2.6.3 (Hyatt et al. 2010) was used to predict putative protein coding sequences and single-copy marker genes were extracted with FetchMG v.1.0 (Kultima et al. 2012). The predicted protein sequences were aligned and classified with DIAMOND v.0.9.29 (Buchfink et al. 2021). Taxonomic classification was done by classifying the contigs based on the predicted rRNA, single-copy marker genes, and total proteins, respectively, using KronaTools (Ondov et al. 2011).

### Fluorescence-activated cell sorting (FACS)

Ten microliters of algal suspension was diluted in 990 µl sterile phosphate-buffered saline (PBS). After filtration through a 20-µM-pore size filter, the samples were analyzed by flow cytometry using a fluorescence-activated cell sorting (FACS) instrument (FACSMelody™, BD Biosciences, San Jose, CA, USA). To this end, the samples were excited at *λ*_Ex_ = 488 nm and *λ*_Ex_ = 640 nm, respectively (Basheva et al. 2018; Stadnichuk et al. 2015). The detection of the fluorescence was performed by recording the emission at *λ*_Ex_ = 783 nm, and the data was analyzed using the software BD FACSChorus™ v. 1.4.3.0 (BD Biosciences, San Jose, CA, USA).

## Results

### Setup of a photobioreactor coupled with a chicken coop

To investigate whether the functional coupling of a photobioreactor with an animal house enables the production of biomass from agricultural emissions, we used a helical tube photobioreactor (Watanabe and Hall 1996) implemented in a suspended structure (Fig. 1), which, under aeration with exhaust air from a chicken house was used for the continuous cultivation of photosynthetic microorganisms (PMO) of the commercially relevant genus *Arthrospira spec*, often referred to as *Spirulina* microalgae (Gonçalves et al. 2016; Soni et al. 2019). As detailed below, quantification of algal biomass, CO_2_, and NH_3_ concentration (Figure S1, Figure S2) revealed that the coupled system is capable of effectively utilizing the exhaust air from the chicken house for resource-efficient and sustainable algal biomass production in a continuous operation.

For testing of the suspended helical cone-shaped photobioreactor, it was connected to exhaust air from a chicken coop via tubing (Fig. 1A). The chicken coop (ca. 16 m^2^, 54 m^3^) housed 50 laying hens and 2 roosters, which during the day had access to an outdoor area through an automatically controlled chicken flap. Details are given in Supplementary Figure S3. A ventilation system extracted exhaust air from the henhouse with a turnover of about 175 m^3^/h, and a part of this exhaust air of about 1 m^3^/h was injected into the lower inlet of the helical tube of the photobioreactor via a pump through a hose connection (green line, in Fig. 1A, B). The CO_2_ concentration in the exhaust air of the chicken house was measured continuously with a sensor (“CO_2_ input,” Fig. 1A). The air blown into the bioreactor tube via an air inlet valve at the bottom rises into the reservoir through the helically wound tube and escapes via the gas outlet after passing through the “CO_2_ output” sensor. The buoyancy of the rising air transports the liquid medium through the helix into the reservoir, from where it flows back through a central return tube into the lower part of the helix (Fig. 1B).

In the reactor prototype used here, the helically wound cultivation tubing consists of polyvinyl chloride (PVC) with an inner diameter of 40 mm and a total length of 100 m. Variants were also tested in which the cultivation tube consisted of two different sections of tubing, e.g., PVC and silicone, connected by flanged collars (lower and upper part of the reactor, respectively, in Fig. 1C) to investigate the variation in bioadhesion of the cultured microalgae to the different materials. The reservoir typically had a volume of 50 l, so that a total of about 145 l of liquid culture medium was moved in the reactor as well as a volume of air of about 35 l. Circulation of the liquid volume was achieved by a continuous air flow of 15 l/min, resulting in typical medium flow rates of about 100 l/h during the cultivation of algae. The external dimensions of the helical cone were approximately 1.15 m and 2.10 m in lower and upper diameter, respectively, and spanned a height of 1.25 m. The entire device was mounted under the ceiling of the room via a suspension device.

An array of 15 LED light bars, each with a power of 28 W, was placed inside the spiral cone to provide light energy to the PMO growing in the reactor. In typical cultivation experiments, the reactor was inoculated with *Arthrospira* sp. microalgae (*Arthrospira platensis*) obtained from an open pond culture of a microalgae farm. Open pond cultures usually are a mixture of microorganisms also containing, for example, diatoms and prokaryotic species. Continuous cultivation in the helical tube reactor was carried out in a culture medium for periods up to 6 weeks, with samples taken at regular intervals to quantify biomass. For this purpose, the optical density at 565 nm was determined and converted to dry mass using a calibration curve (Figure S1). Typically, every 2–4 days, the algal biomass had a sufficiently high density to be harvested by rotational filtration.

### Continuous cultivation of *Arthrospira* microalgae

**Fig. 2:**
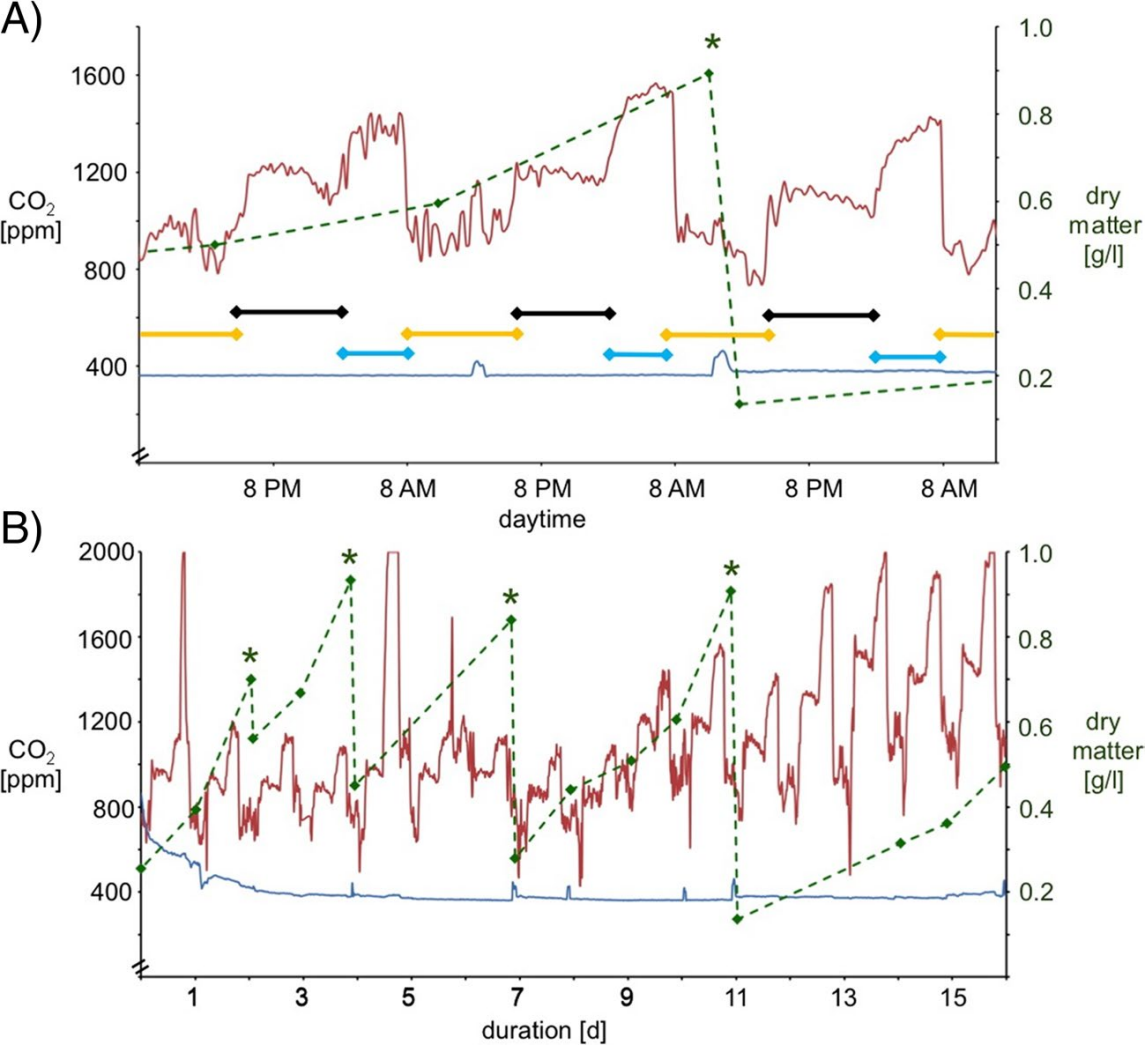
Cultivation of *Arthrospira* sp. microalgae with exhaust air from chicken coop. **A** Representative plot of CO_2_ concentration in the daily cycle of the chicken house (CO_2_ input, red graph) fed into the algae reactor. The blue graph shows the CO_2_ concentration of the exhaust air after passage through the algae reactor (CO_2_ output). The colored bars show the daytime phases with active chickens when the outer flap is open (yellow), sleeping chickens when the flap is closed (black), and in the morning when the outer flap is closed and the chickens are awake in the coop (blue). The dashed green line represents the algae biomass in the reactor, and the harvest is marked by a green asterisk. **B** Representative data on CO_2_ turnover and biomass production in the reactor over a 16-day period. Data were collected in mid-November when the barn had a daylight cycle from 5 AM to 6 PM due to artificial lighting. Same color coding for the graphs as in (**A**). Note that in the course of this experimental series, varying amounts of biomass were removed on the days indicated by green asterisks

To obtain quantitative data on CO_2_ turnover and biomass production in the helical cone reactor (Fig. 2), the CO_2_ concentration in the chicken house and thus in the CO_2_ input line was continuously monitored. The CO_2_ concentrations in the chicken house (red line, Fig. 2A) followed a characteristic pattern based on the day/night activity of the chickens and the opening of the outer flap. When the flap was open and the chickens were allowed into the outside enclosure (yellow slots, Fig. 2A), the lowest CO_2_ levels of about 900 ppm were measured. The CO_2_ concentration increased sharply when the chickens gathered in the coop towards evening and the flap was closed (about 1200 ppm), and it then decreased by about 150 ppm during the night rest (black slots). In the morning, when the chickens were active in the coop with the flap closed (blue slots), the CO_2_ level increased to peak values around 1600 ppm. Strikingly, the CO_2_ concentration in the air after passing through the algae reactor shows no such pattern, but rather a nearly constant level of about 400 ppm (blue line, in Fig. 2), which corresponds to the CO_2_ concentration of outdoor air (about 330–400 ppm). A further drop below 400 ppm was not observed, presumably because the algae do not fix significantly more CO_2_ below this level and/or because the exhaust air from the reactor could not be strictly separated from the ambient air.

Figure 2B shows a representative data set for long-term monitoring of CO_2_ turnover (red and blue plots, in Fig. 2B) and biomass production (green dashed line) in the helical cone reactor. At the start of cultivation (day 1), when the freshly prepared medium was inoculated with *Arthrospira* sp. in the algae reactor, the red CO_2_ input and blue CO_2_ output curves both showed approximately the same value of about 750 ppm. As the algal biomass increased within the first 3 days, the CO_2_ output then leveled off to the value of about 400 ppm already discussed in Fig. 2A. It is noteworthy that the harvests performed during cultivation, in which variable portions of the medium were cleared of microalgae by rotational filtration on days 2, 4, 7, and 11, resulted in almost no increase in CO_2_ output signal. Only very small increases could be temporarily observed after harvesting on days 7 and 11. Also, approximately 25% reduction in stall ventilation, applied from day 10 to increase CO_2_ input, did not result in a significant increase in the output signal. Control measurements during routine operation of the algae reactor confirmed proper functioning of the CO_2_ monitoring system (Figure S4), and more detailed measurements at an overall higher CO_2_ input from the chicken house showed that the CO_2_ output was indeed correlated with the amount of algal biomass in the reactor (Figure S5). Furthermore, monitoring of NH_3_ concentration using colorimetric test strips showed that the average concentration of about 35 ppm in the chicken house exhaust air was efficiently reduced to about 5 ppm after passage through the algae reactor (Figure S2).

The data shown in Fig. 2B also allow an estimate of productivity, although the prototype system studied here has not yet been optimized. For example, the growth rates shown as a green curve suggest that the reactor can be operated for the algae are in the exponential growth phase (e.g., see days 2–5 or 9–12). Under these conditions, typical production rates of greater than 0.3 g/l*day dry mass were obtained (Figure S6).

### Characterization of the *Arthrospira* microalgae culture

The *Arthrospira* microalgae culture used in this pilot study was characterized in terms of physiological performance and microbiological composition. The evolution of the pH of the culture medium followed the expected course since the initial pH of the medium increased from 9 at the beginning over the course of cultivation to pH 11 at high biomass concentrations, but generally averaged around pH 10 (Figure S7).

**Fig. 3:**
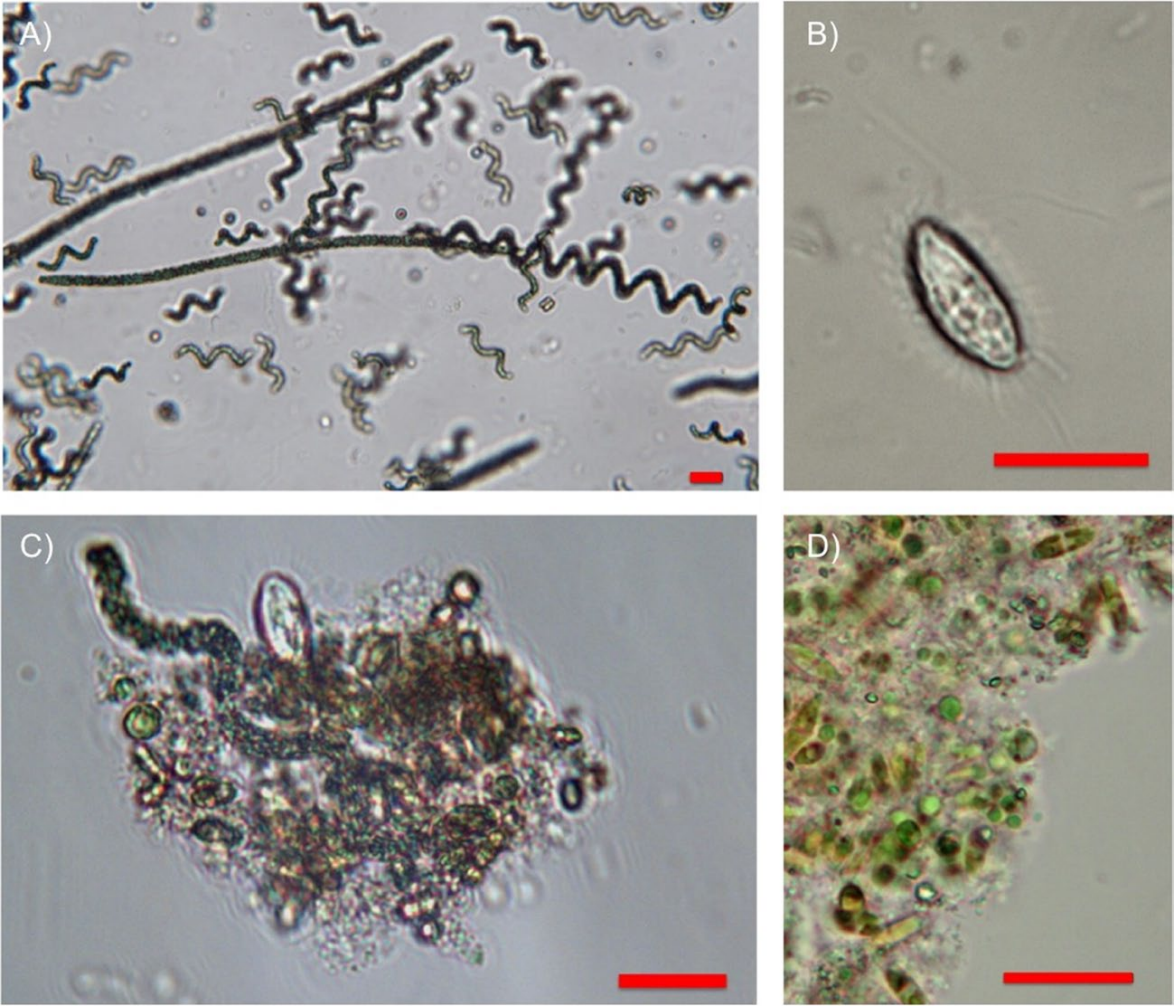
Light microscopy images of the *Arthrospira* sp. microalgae culture used in this study. **A** Overview image of cell culture suspension, revealing primarily helical and straight *Arthrospira* as well as small microorganism, e.g., **B** ciliates. **C**, **D** Cell aggregates from various microbes revealing phytoplankton such as diatoms and *Nannochloropsis*. Scale bars are 20 µm

The microbial composition of the *Arthrospira* microalgae culture was also investigated by light microscopy (Fig. 3). The overview image (Fig. 3A) shows the predominant occurrence of *Arthrospira* in the mixed culture, which are present in both the typical helical and straight elongated filamentous phenotype. The occurrence of both spiral and straight phenotypes, both showing the typical intracellular cross-walls of *Arthrospira* (Figure S8), suggested that different strains were present in the mixed culture (Mühling et al. 2006). However, a variety of other microbial organisms were also observed, including phytoplankton with diatoms, *Nannochloropsis*, and ciliates, as well as species that could not be clearly assigned microscopically (Fig. 3B–D). In addition, a variety of bacteria were visible only as small, highly mobile particles with weak contrast at higher magnification (Figure S8). To further specify these morphological studies, we performed metagenomics analyses of samples of mixed *Arthrospira* microalgae culture taken before and after long-term cultivation.

**Fig. 4:**
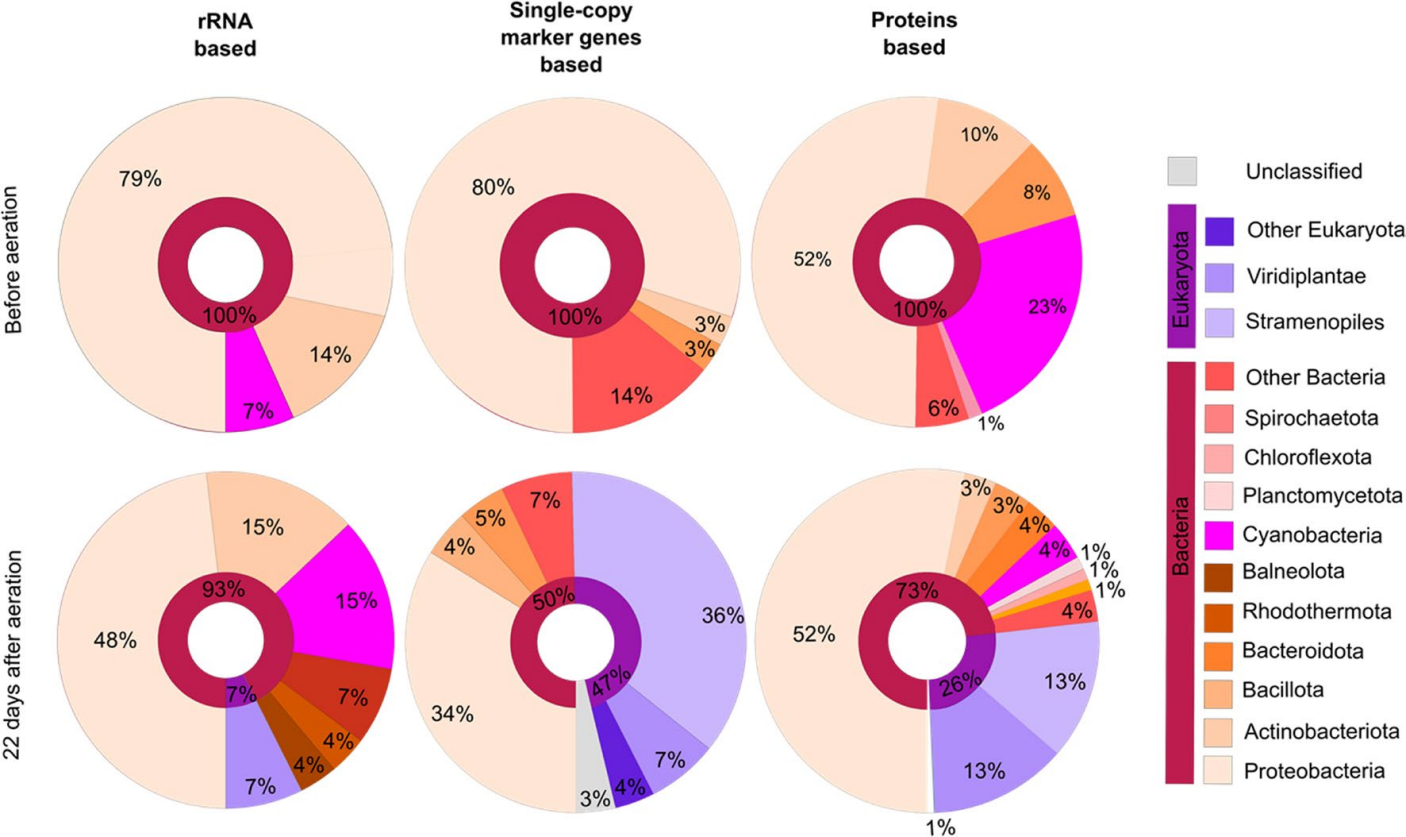
Taxonomic distribution of the algae suspension samples before and 22 days after the algae culture was aerated with exhaust air from the chicken coop. The taxonomic classification was calculated based on rRNA, single-copy marker genes and total proteins, respectively. The results were visualized based on their taxonomy using KronaTools

To this end, samples from the reactor were sequenced on an Illumina NextSeq550 platform and the resulting metagenomic sequences were processed, merged, and assembled. Coverage profiles were created, which were used to perform the taxonomic classification based on rRNA genes, single-copy marker genes, and total proteins, respectively. The resulting taxonomic classification shows that before aeration (Fig. 4, first row), the community was composed of primarily bacterial organisms, while after the reactor was exposed to exhaust air from the chicken coop (Fig. 4, second row), the microbial composition changed, and eukaryotic species could be detected. The taxonomic classification resulting from all three analysis methods showed that the initial culture was composed of *Proteobacteria* (Fig. 4, light orange), *Actinobacteriota* (Fig. 4, orange), and *Bacteroidota* (Fig. 4, dark orange). Additionally, the analysis based on rRNA genes and proteins show 7% and 23% of *Cyanobacteria* (Fig. 4, pink), respectively, thereby revealing members belonging to the genus *Arthrospira* (not shown). After aeration, the community composition within the bacterial kingdom showed a shift to a more diverse microbial community. Here, the emergence of members of the eukaryotic kingdom after the exposure of the reactor to exhaust air is noteworthy. The appearance of *Viridiplantae* (Fig. 4, violet), which represent at least 5% of the overall community, includes organisms belonging to the genus of *Chlorella* (data not shown). Moreover, organisms belonging to *Stramenopiles* (Fig. 4, light violet), which include diatoms (Fig. 3B), were apparently enriched by exposure to exhaust air from the chicken coop.

It is important to note that, while the sequencing results only show the presence of *Cyanobacteria* max. 23% before (Fig. 4, first row, pink) and 15% abundance after aeration (Fig. 4, second row, pink), this does not translate into the overall composition of the biomass. The microscopy images (Fig. 3) show that cyanobacterial cells are significantly bigger than other microorganisms, which can only be identified in the background (Figure S8). These differences in cell size lead to the majority of the biomass being *Cyanobacteria*. To further verify the presence of *Cyanobacteria*, samples were analyzed using fluorescence-activated cell sorting (FACS) to detect their autofluorescence (Figure S9). The results confirmed that *Cyanobacteria* comprise the majority of biomass in the algal reactor after 22 days of being aerated with the exhaust air.

## Discussion

To investigate the performance of a suspended helical cone photobioreactor for reducing gas emissions from a chicken house to produce *Spirulina* (*Arthrospira spec.*) *Cyanobacteria*, the exhaust air from a chicken coop was continuously monitored in terms of CO_2_ and NH_3_ concentration. We found a significant correlation of diurnal activity and sleep rest of the chickens with CO_2_ production, which is consistent with diurnal variations documented under normal agricultural conditions (Pedersen et al. 2008). Both CO_2_ and NH_3_ concentrations were significantly reduced after the exhaust air passed through the photobioreactor. The air monitoring data clearly indicated that the algae reactor acts as an effective biofilter for the exhaust air from the chicken house and can convert it into valuable *Arthrospira* biomass.

The unconventional shape of our photobioreactor of a free-hanging inverted helical cone takes into account that conventional barns require the entire floor space for the animals living in the barn (e.g., poultry, pigs, cattle, etc.). Therefore, a photobioreactor installed directly in the barn should not only have the smallest possible footprint but also have high physical robustness to avoid mechanical damage to the reactor and its operating elements (e.g., light fixtures, pumps) due to accidental contact with animals or farm personnel. The here described prototype offers high physical robustness, and with its low space requirement due to the suspended installation of 0.17 and 0.05 m^3^ of culture volume per m^2^ of footprint occupied by the lower and upper sections of the reactor, respectively, the not yet optimized reactor offers a surface-to-volume ratio of about 100 m^2^/m^3^ to enable continuous cultivation of the *Arthrospira* mixed culture.

As expected, morphological characterization of the *Arthrospira* mixed culture by light microscopy revealed the predominant presence of *Arthrospira* as well as a variety of other microbial organisms, including phytoplankton. Metagenomics analyses of the mixed culture before and after long-term cultivation with chicken exhaust showed a shift towards a more diverse microbial community. Diversification after aeration suggested a synergistic effect between the algal reactor and the chicken house environment. The appearance of organisms belonging to the genus of *Chlorella* after the aeration may explain how the microbial community adapts to the new environment, as organisms belonging to this genus have shown a great tolerance against NH_3_, present in the exhaust air (Figure S2), and have previously been found in contaminated wastewater plants (Salbitani and Carfagna 2021; Wang et al. 2018). Moreover, organisms belonging to *Stramenopiles* (Fig. 4, light violet), which include diatoms, were apparently enriched by exposure to exhaust air from the chicken coop. Diatoms possess the ornithine-urea cycle (OUC) to metabolize NH_3_ and can therefore optimize nitrogen management in their ecosystems (Horák et al. 2020; Smith et al. 2019). The rigid OUC cycle allows diatoms to efficiently adapt to a changing nitrogen status of their environment (Smith et al. 2019).

Closed photobioreactors on a commercial scale have not been widely reported in the scientific literature (Kunjapur and Eldridge 2010) making it difficult to rank the performance of the helical cone reactor described here. In the early 2000s, the 700-m^3^ tube production plant in Klötze, Germany, was considered the world’s largest example of a closed reactor system, for which a review paper reported a production rate of *Chlorella vulgaris* of 130–150 tons dry biomass/year (Spolaore et al. 2006). It is known that *Chlorella* can show higher growth rates than *Arthrospira* (Gonçalves et al. 2016; Mata et al. 2010) and optimized growth rates of only about 0.08 g/l*day dry mass have recently been reported for *Arthrospira* at laboratory scale (Soni et al. 2019). Furthermore, a greenhouse-operated 1000 l photobioreactor yielded an average productivity of 0.055 mg/l*day *Arthrospira* biomass (Delrue et al. 2017). Thus, the typical production rates of about 0.14 g/l*day dry biomass determined in this work for the helical tube reactor operated with chicken house exhaust air are at least competitive with these literature-described performances.

The chemical process of the simultaneous absorption of CO_2_ and NH_3_ in water has long been studied (Pinsent et al. 1956). Due to the high solubility of NH_3_ and the relatively low solubility of CO_2_ in water, the concentration of NH_3_ in water is higher than that of CO_2_. The reaction between NH_3_ and CO_2_ in water can therefore be described as follows:

$$\mathrm {CO}_{2} + 2\;\mathrm{NH}_{3} =  \mathrm{NH}_4^+ +  \mathrm{NH}_{2} \mathrm{COO}^{-} .$$

The reaction between NH_3_ and CO_2_ in water has been shown to be rapid and a pseudo-first-order reaction, and CO_2_ is completely consumed at the gas/liquid interface due to the lower solubility of CO_2_ compared to NH_3_ (Pangarkar 1974). However, accurate quantification of the volumetric mass-transfer coefficients (KGa) of CO_2_ in NH_3_ and CO_2_ absorption in water requires specific experimental and theoretical models. In the case of a specific rotating packed-bed reactor system, for example, the KGa of CO_2_ was found to be 2–6 times higher when NH_3_ and CO_2_ were absorbed simultaneously than when CO_2_ was absorbed alone (Sun et al. 2012). Although KGa values cannot be easily quantified for the system described here due to the high complexity resulting from the nutrient medium, the irregular flow profile of the air bubbles, and the dynamic growth of the biomass, these basic processes should also apply.

Due to the rapid absorption of CO_2_ and NH_3_ (Pangarkar 1974; Pinsent et al. 1956), the slow flow rates in our reactor and the resulting long gas/liquid contact times, and the fact that we observe constant rates of decrease in CO_2_ and NH_3_ concentration in the exhaust air before and after its passage through the reactor over long periods of time (Fig. 2, Figure S2), the values obtained in this study can be used for quantitative consideration. In the above experiments, an average continuous absorption of about 700 ppm CO_2_ and 35 ppm NH_3_ was observed, leading to an average productivity of 0.13 g/l*day and peak productivities of 0.3 g/l/day biomass. During routine daily operation of the reactor, however, typically absorption of about 1200 ppm CO_2_ and 35 ppm NH_3_ is observed, leading to an average productivity of 0.14 g/l/day and peaks of 0.4 g/l/day biomass (data not shown). Hence, we do not observe a linear relation between CO_2_ absorption and productivity, suggesting that carbon is not the limiting factor for biomass growth in the current setting. Assuming optimization of other parameters, such as light energy input, as well as typical CO_2_ concentrations (≤ 3000 ppm) in agro-industrial chicken houses (Grote et al. 2006), we estimate that about 2500 ppm CO_2_ can be absorbed with the reactor, leading to average production rates of about 0.3–0.5 g/l*day biomass.

While all of the above productivities can easily compete with a greenhouse-operated 1000-l photobioreactor that conventionally used carbonate to deliver an average productivity of 55 mg/l/day *Arthrospira* biomass (Delrue et al. 2017), our method can also be evaluated in terms of carbon capture and utilization (CCU) technologies. At a volumetric flow rate of animal house exhaust air of 21.6 m^3^/day (15 l/min) with a routine absorption of 1200 ppm CO_2_ and 35 ppm NH_3_ and the density of CO_2_ and NH_3_ (1 ppm corresponds to 1.96 and 0.76 mg/m^3^, respectively), the prototype described here captures about 50 g/day and 576 mg/day of CO_2_ and NH_3_, respectively, which are absorbed in the liquid phase and eventually incorporated into the biomass. The reactor is operated with an energy input of about 11 kW/d (420 and 40 watts for lighting and pumps, respectively), to achieve the average productivity of about 20 g/day (0.14 g/l*day) of biomass, thus showing a current efficiency of about 550 Wh/g biomass. With regard to CCU, the CO_2_ consumption required for generating electrical energy must be included. According to statistical surveys of the German Federal Environmental Agency, about 420 g CO_2_ per kWh was needed for this in 2021 (Icha and Lauf 2022). Based on this figure, our pilot plant invests about 230 g CO_2_ in electrical energy to capture about 2.5 g waste CO_2_ and produce 1 g *Arthrospira* biomass. However, it must be taken into account that the CO_2_ balance would be significantly better if renewable energy sources, such as wind and solar power, were used to operate the reactor. In this respect, it was estimated that an efficiency of 3.5 Wh/g *Spirulina* biomass can be achieved with CO_2_-free generated light energy (Delrue et al. 2017). Furthermore, our approach brings the additional benefit from the sequestration of waste NH_3_, which would otherwise have to be purchased as bioavailable nitrogen for algae cultivation and remunerated as a pollutant emission of an agricultural plant.

Since both energy prices and financial returns for the biomass produced are subject to strong regional and temporal fluctuations, no estimate of the monetary balance can be made at this point. However, it should be emphasized that scaling up to larger reactor volumes would significantly improve the above estimates. For example, we assume that by increasing the pitch and number of turns of the helix, a tenfold increase in the reactor volume should be easily feasible, resulting in higher gas volume flows processed, better light utilization, and higher production efficiencies without significantly increasing the footprint. Similarly, other process parameters such as composition of culture media and microbial communities can be optimized in a variety of ways to improve bioproduction processes in the reactor and thus to create new opportunities for the production of value-added products from gaseous CO_2_ and NH_3_ waste emissions. We would also like to note that the aspect of economic efficiency of operating costs and additional capital investments, which was often critically questioned in the past, will be increasingly extended in the future to include the aspect of sustainability of the production processes due to climate change and the resulting problems. Here, the presented approach offers unique advantages, as the resource-saving production of microalgae is combined with the simultaneous sequestration of animal emissions.

In conclusion, we presented the functional coupling of a photobioreactor with an animal house, enabling the production of biomass from agricultural emissions. While the use of microalgae cultivation to recycle nutrients from industrial emissions, such as flue gas, has been studied (Cheah et al. 2015; Kumar et al. 2011), the present work is, to the best of our knowledge, the first practical demonstration that this approach can also be used to recycle gaseous emissions from animal housing, paving the way for sustainable approaches to a circular economy in animal agriculture.

### Supplementary Information

Below is the link to the electronic supplementary material.

Supplementary file1 (PDF 661 KB)

## Data Availability

The authors declare that the data supporting the findings of this study are available within the paper and its Supplementary Information files. Sequence data from Illumina sequencing are available at BioProject PRJNA979969. Should any raw data files be needed in another format, they are available from the corresponding author upon reasonable request.
