# Supplementary material for: A photobioreactor for production of algae biomass from gaseous emissions of an animal house
Source: Appl Microbiol Biotechnol. 2023 Oct 10;107(24):7673–84. doi: 10.1007/s00253-023-12815-7 (PMC10656320; doi:10.1007/s00253-023-12815-7)
Supplement: Supplementary file 1 — Supplementary file1 (PDF 661 KB) [file 253_2023_12815_MOESM1_ESM.pdf]

Applied Microbiology and Biotechnology

A photobioreactor for production of algae biomass from gaseous emissions of an animal house

Till Glockow[a], Marta Velaz Martín[b], Laura Meisch[b], Denis Kapieske[a], Kai Meissner[a], Maximiano Correa Casal[c], Anne-Kristin Kaster[c], Kersten S. Rabe[b], and Christof M. Niemeyer[b*]

[a] Acheron GmbH, Auf der Muggenburg 30, D-28217 Bremen, Germany

[b] Karlsruhe Institute of Technology (KIT), Institute for Biological Interfaces 1 (IBG-1), Biomolecular Micro- and Nanostructures, Hermann-von-Helmholtz-Platz 1, D-76344 Eggenstein-Leopoldshafen, Germany.

[c] Karlsruhe Institute of Technology (KIT), Institute for Biological Interfaces 5 (IBG-5), Biotechnology and Microbial Genetics, Hermann-von-Helmholtz-Platz 1, D-76344 Eggenstein-Leopoldshafen, Germany.

*Email: christof.niemeyer@kit.edu

Telephone: 0721 608-2-3000

Fax: 0721 608-2-5546

Supporting Information

Supplementary Figures

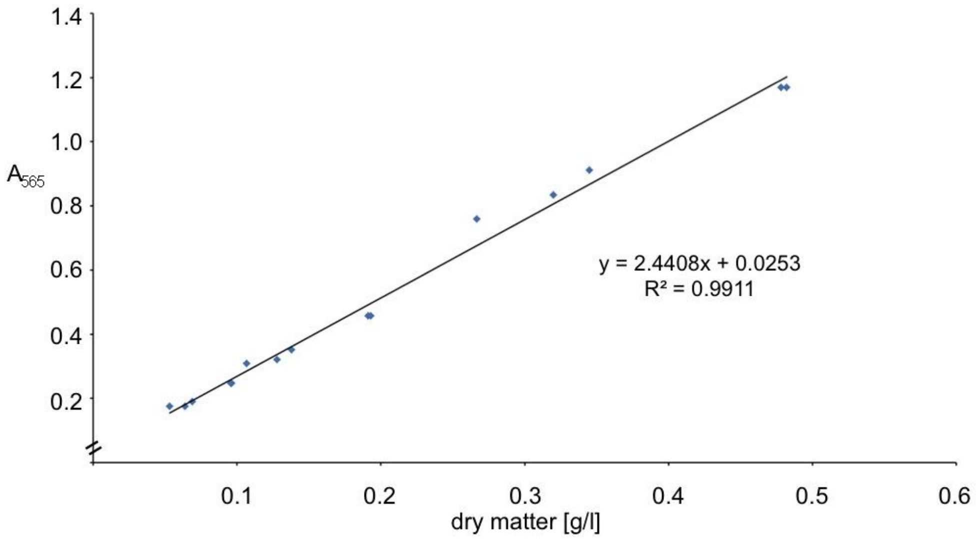

Supplementary Figure S1: Calibration curve for determining dry mass of *Athrospira platensis* culture using the optical density at 565 nm.

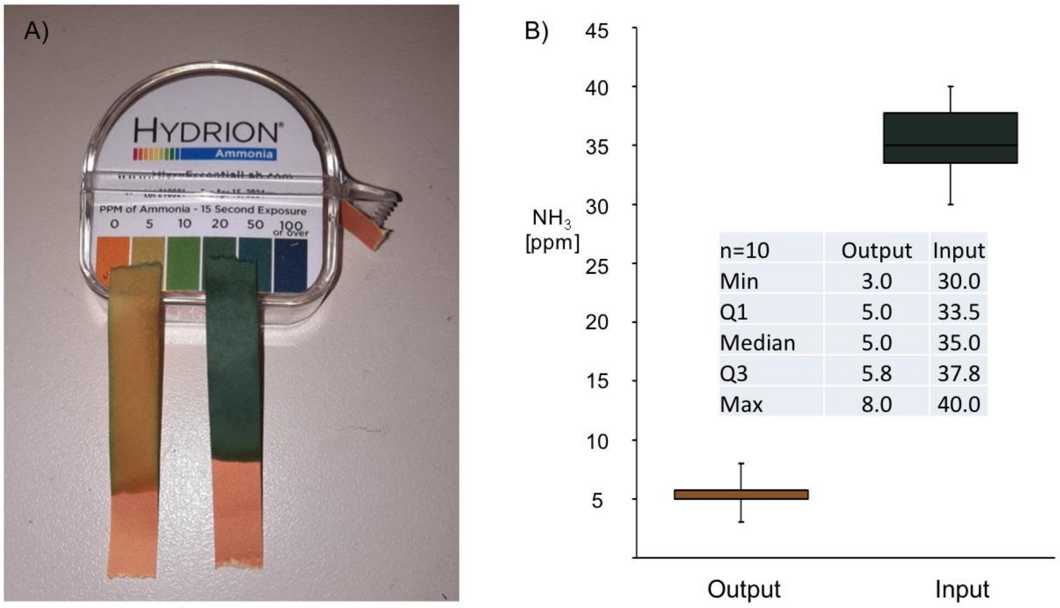

Supplementary Figure S2: Quantification of NH_3 in the exhaust air of the chicken house before (input) and after (output) passage through the algae reactor. A) Photographic image of the Hydrion Ammonia Test Paper strips that were placed in the gas streams along with the color scale on the test kit. B) Statistical analysis of individual NH_3 concentrations determined over a longer period of time (> 30 days) from various data points.

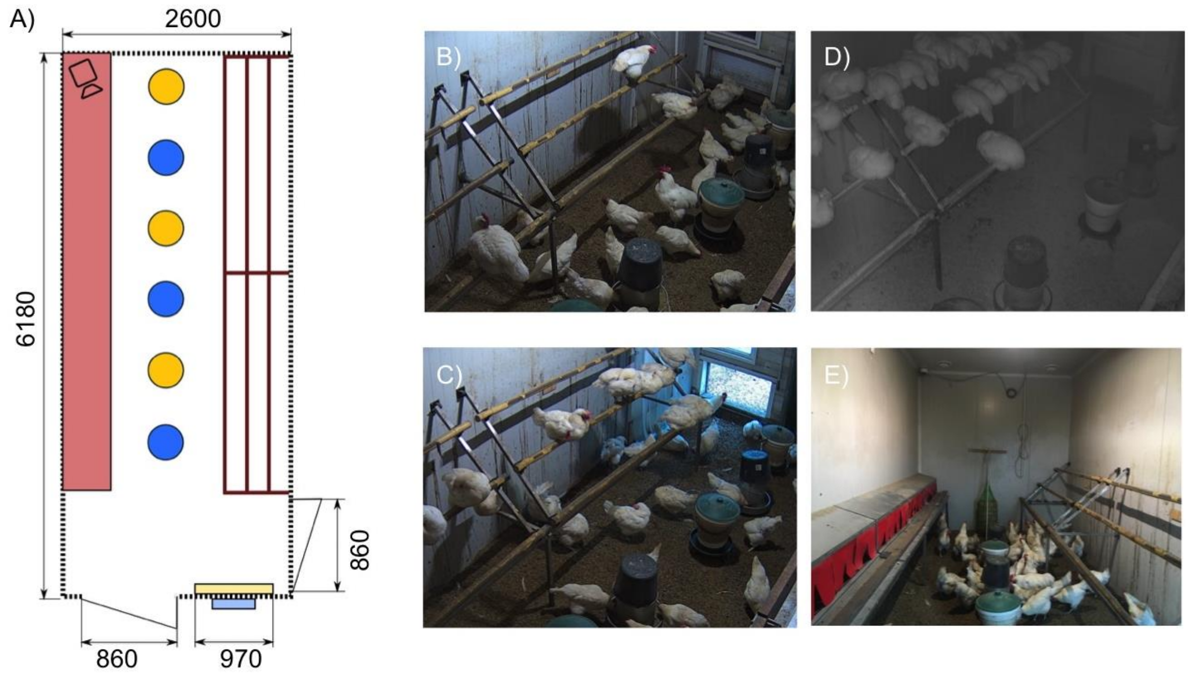

Supplementary Figure S3: Details of the chicken coop. A) Schematic diagram of the coop with dimensions, all numbers given are in mm, the room height is 3370mm. The red box on the left and the grid on the right indicate the laying nests and perches, respectively. The circles in the middle are containers for feed (orange) and water (blue). The computer-controlled chicken flap (970 x 450 mm) can be seen at the bottom right (yellow box), the position of the web camera is at the top left under the coop ceiling. Images from the web camera at different times of the day show the phase with active chickens when the flap is closed (B), the day's activity when the flap is open (C) and the night rest (D). The chicken flap can be seen in the top right of the images. The image in D) shows the coop from the opposite perspective during the day.

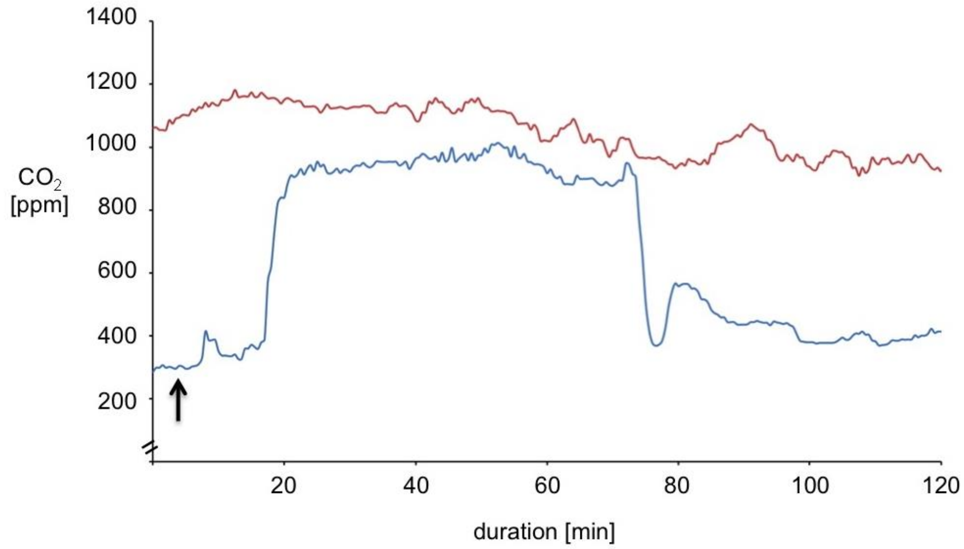

Supplementary Figure S4: Plot of CO_2 concentrations measured during routine operations on the algae reactor. The red graph shows the CO_2 input that was fed from the chicken house into the algae reactor during a typical day, the blue graph shows the CO_2 concentration of the exhaust air after passing through the algae reactor. At about 5 min (marked by arrow), the entire volume of liquid was removed from the algae reactor, causing the blue curve to rise to the level of the red curve. After refilling, the blue output value droped again to the original value of approx. 400 ppm.

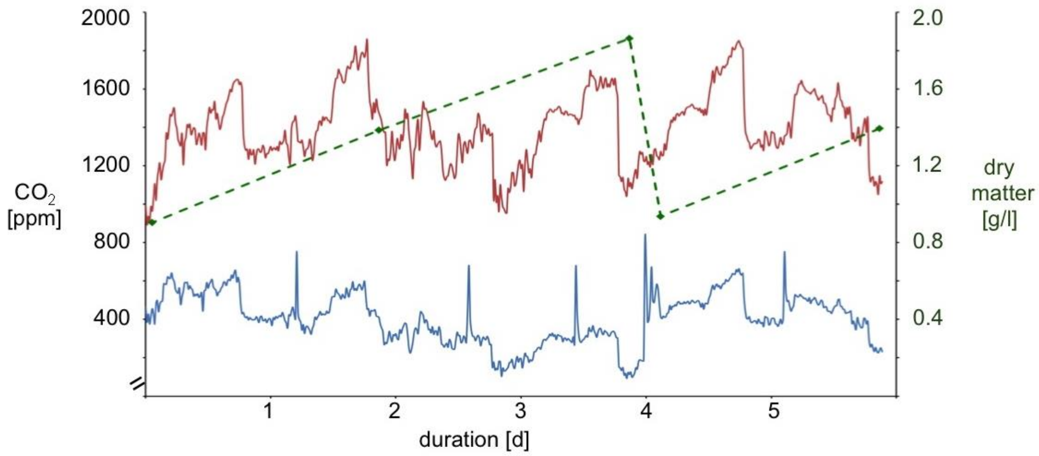

Supplementary Figure S5: Detailed measurements of CO_2 input from the chicken house (red graph) and CO_2 output after passing through the algae reactor (blue). Note the progressive decrease in the level of the output signal with increasing biomass from day 1 - 4 as well as the renewed increase on days 4 – 5 after removal of the algal biomass on day 4.

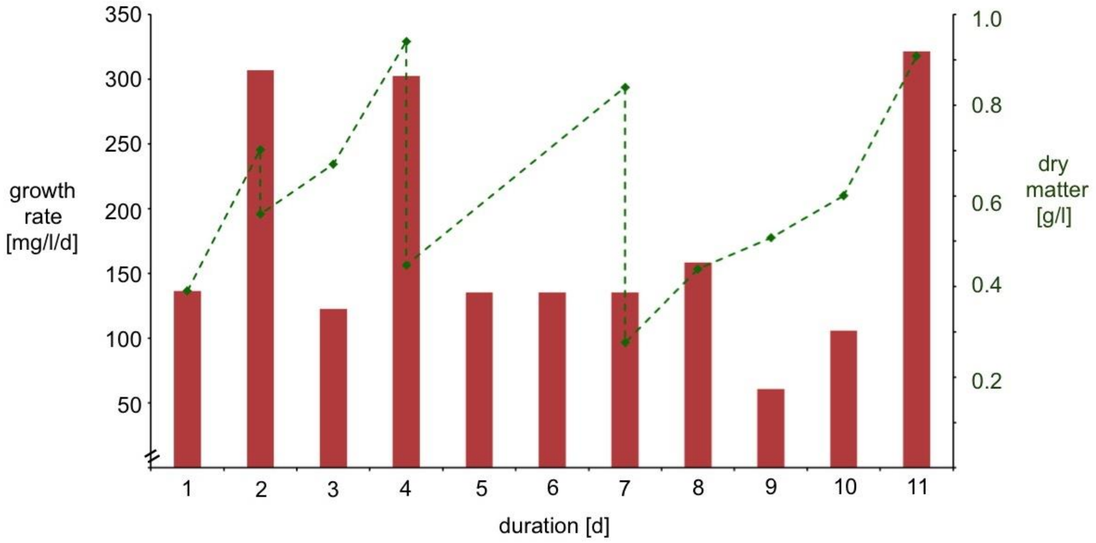

Supplementary Figure S6: Representative growth rates during continuous cultivation of *Arthrospira* microalgae. The green graph shows the progression of biomass over the cultivation time, the red bars show the calculated growth rates.

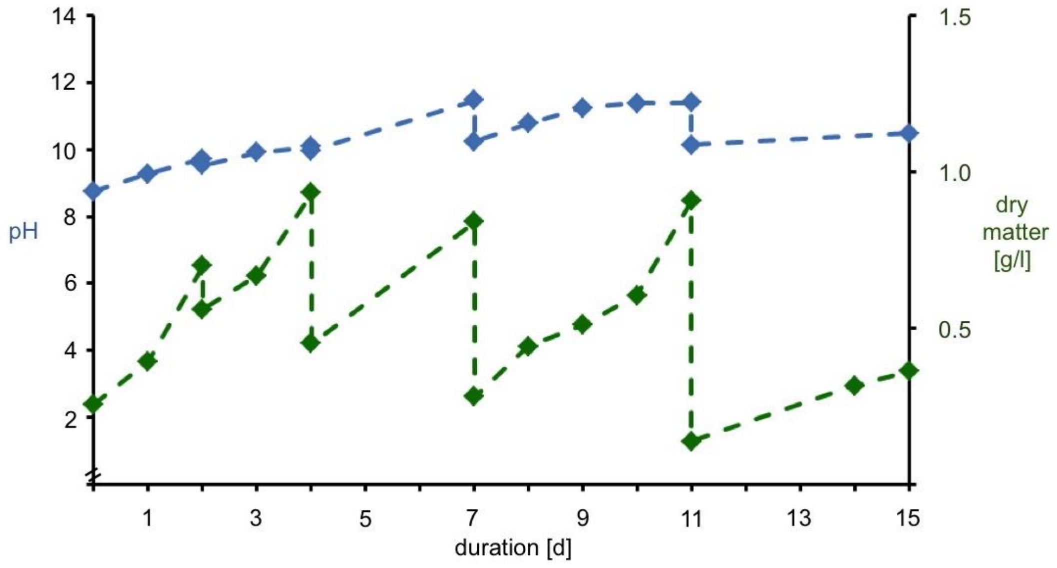

Supplementary Figure S7: Representative course of the pH value of the culture medium in the course of continuous cultivation of *Arthrospira* microalgae. The blue and green graphs show the evolution of pH and biomass, respectively, over the cultivation period.

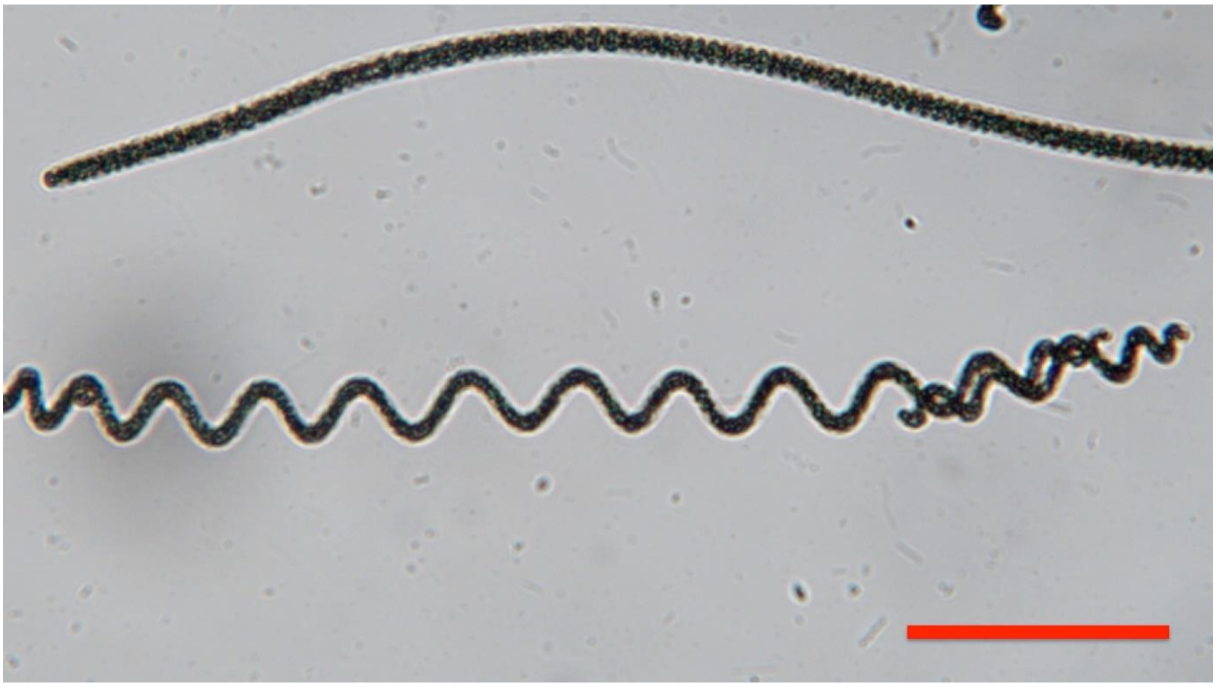

Supplementary Figure S8: Light microscopy image of spiral and straight *Arthrospira* phenotypes present in the mixed microalgae culture used in this study. Note the various small (sub)micrometer sized particles with weak contrast that appear blurry due to their high mobility, which are presumably bacteria. Scale bar is 100 µm

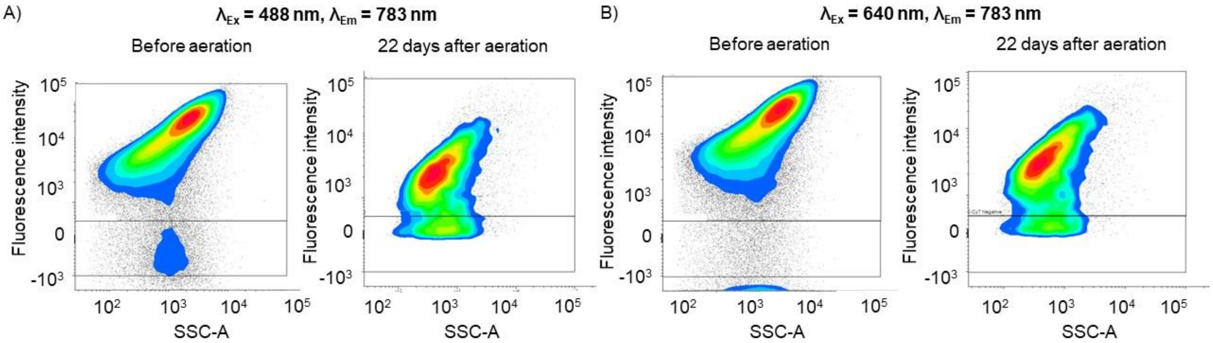

Supplementary Figure S9: Fluorescence of samples before and after being exposed to the exhaust air of a chicken coop. Due to the complex nature of the sample, many chromophores are present and as a result many FRET combinations are possible and therefore the samples were excited at (**A**) λ_{Ex}=488 nm and (**B)** λ_{Ex}=640 nm to detect the autofluorescence of cyanobacteria, which is mediated by phycoerythrin, allophycocyanin, and other proteins that belong to the phycobiliproteins and can form a complex with phycobilin, which acts as a chromophore to capture light energy and transfer it to chlorophyll during photosynthesis (Basheva et al. 2018; Stadnichuk et al. 2015). The emission was recorded at λ_{Em}=783 at λEm=783±28 nm, which covers the range of bacterial chlorophylls and thus should be the final FRET acceptor. Due to the complexity of the community analyzed autofluorescent cells of many sizes, as indicated by the side scatter (SSC-A), can be detected. The number of cells is represented by a color gradient starting with blue (low) to red (high).
